# Students as Partners. Implementation of Climate Change Education Within the Harvard Graduate School of Education

**DOI:** 10.1007/978-3-030-57927-2_6

**Published:** 2020-12-04

**Authors:** Annie Hyokyong Nam, Sueyoon Lee

**Affiliations:** Harvard Graduate School of Education, Harvard University, Cambridge, MA USA; Harvard Graduate School of Education (HGSE), Cambridge, MA USA

## Abstract

This chapter notes the efforts of implementing a climate change curriculum within the Harvard Graduate School of Education that helps to build competencies for potential leaders in different education sectors so that they can collaboratively combat climate change. Literature points out the fruitful and productive partnerships of grassroots initiatives with large scale institutions and/or government organizations. The authors explore the conception of a climate change curriculum with explicit content knowledge and thoughtful pedagogy, designed by students and supported by faculty. The authors examine the design elements of the curriculum and then specify the implementation process of a curriculum at the Harvard Graduate School of Education (HGSE). The authors draw out the limitations and implications of “students as partners” in the co-creation of learning and teaching in the field of sustainable development education within higher education institutions.

## Beyond the Bottom-Up and Top-Down Debate on Climate Change Education

Climate is a dynamic interplay of the atmosphere, hydrosphere, cryosphere, biosphere, and lithosphere (Aspen Global Change Institute 2019). Whereas weather is defined by fickle fluctuations from day to day, climate change is a long term, sustained trend of change in climate. As rising anthropogenic greenhouse gas emissions continue to trap heat in the form of infrared radiation (Fahey 2012), global warming has been occurring at rates much faster than anticipated and its effects being clearly felt worldwide.

Despite alarming amount of evidence from the scientific community, global warming remains an issue of political debate in the United States, accentuating the ideological phenomenon categorized as ‘climate change denial (Hess and Collins 2018).’ The withdrawal of the United States from the 2015 Paris Agreement Treaties (Pompeo 2019) and the reversal of numerous environmental rules and regulations (Clements et al. 2020) indicate that American politics may stand in the way of achieving an environmentally sustainable future. Consequently, local and regional actors in the US are increasingly stepping forward to fill the policy void created by federal inaction (Reeves et al. 2014). Inciting and mobilizing citizen action could be central in mitigating the effects of climate change (Wi 2019).

If students are educated to understand and act upon changes in climate, they can create a grassroots movement that produces systemic changes. A grassroots movement refers to an initiative to help individuals engage in community interventions and activities with the mission of instrumenting local and societal change for the collective interest of the community (de Souza 2007; Fisher 1998; Rothman 1996). Grassroots organizations have shown promising results in raising awareness and encouraging community involvement (Christens 2010; People’s Association [PA] 2011; Paul and Tan 2003; Smith 2000), but the debate over top-down versus bottom-up approaches in climate change education (CCE) has long persisted (Fadeeva et al. 2014). 

In essence, the main aim of climate change education (CCE), which is rooted in education for sustainable development (ESD) (UNESCO 2015), is to engage different stakeholders in promoting lifelong education for global citizenship and help build a knowledge society in which local communities act upon recommendations. Educating for this type of change is a challenge in traditional western education because education is no longer considered a top-down transmission of knowledge, information, and values (Varga et al. 2007). Rather, a bottom-up approach promotes creativity and ownership of joint involvement and action, allowing for a leveraging of specialized knowledge and experiences contributed by citizens (Fraser et al. 2006; Wi 2019). However, there are some advantages to a top-down educational approach. According to Wi (2019), its simplicity and efficiency in decision making as well as its ability to mobilize more resources and generate widespread awareness can help get things done. Ultimately, Wi argues for a collaboration and infusion of both approaches as key to a successful implementation of CCE.

Literature additionally points out the potentially fruitful and productive partnerships of grassroots initiatives with large scale institutions and/or government organizations. Reeves et al. (2014) suggests governments should set up grassroots initiatives themselves ‘from the outside’ to generate climate change activism among citizens. Within higher education institutions, Fadeeva et al. (2014) talks about the need to move beyond the top-down versus bottom-up participation and embrace a participatory democracy in which everyone is involved and held responsible. Brundiers et al. (2014, p. 196) comment that to move beyond the inertia of academic institutions and break established patterns requires an innovative, multilateral relationships between faculty, students, and surrounding communities.

In this chapter, we explore the question acutely posed by Van der Leeuw et al.

Academic institutions remain so inertial because the professoriate remains in familiar and comfortable patterns. This is human nature but denudes the academy of the energy and passion needed for change. A more bilateral relationship between faculty and students might produce different outcomes. *If students played an equal role in the development of curricula, selection of course content, and initiation of applied projects, how different might the impact of the academy become?* (Van der Leeuw et al. 2012, p. 118)*.*

We begin by examining the necessary design elements of embedding CCE within schools of education and then articulate the implementation process of a CCE curriculum at the Harvard Graduate School of Education (HGSE). Last, we discuss the limitations and implications of “students as partners” in the co-creation of learning and teaching in higher education institutions and in the field of sustainable development education.

## What to Consider When Integrating Climate Change Education (CCE) Within Schools of Education

Previous efforts to embed CCE within education systems have been centered around teacher education (Vega-Marcote and Varela-Losada 2016; Hopkins and McKeown 2014; Varga et al. 2007) or a school-based implementation of a whole school approach (UNESCO 2014). However, neither teachers nor schools are the only constituents of education. Multiple key stakeholders in education such as educational policy makers, researchers, teacher certification boards, teacher education institution executives, administrative staff, and students must all work together in the reorientation towards sustainability. Ferreira et al. (2007) call this a “Whole-of System Model,” in which all elements of the system are engaged in the process of embedding a new curriculum, new policy, or whatever is (collectively) determined to be most needed within the particular contexts.

The difficulty of serving the whole of the education system (Vega-Marcote and Varela-Losada 2016) has long been cited as an existing tension plaguing the current environmental and sustainability education efforts in pre-service and in-service teacher education. Graduate schools of education are hubs that attract pre-professionals and professionals from various roles in education. A CCE curriculum that facilitates the collaboration of pre-professionals and professionals across the education sector would promote peer-learning and collaboration, infusing the diversity of perspectives and modeling a whole-of systems approach.

To note, the desire for a curriculum like ours arose not because we wanted to discourage or diminish the existence of CCE curriculum already blended implicitly within several existing structures in schools of education (Denby and Rickards 2016; Molthan-Hill et al. 2019), but to draw out an explicit curricular experience of CCE and model a new multi-lateral approach among students at schools of education. Too often, when students engage in classes in which elements of sustainable development (SD) are implicit within the content or pedagogy of the class (Denby and Rickards 2016), key competencies in sustainable development (SD) are passed down in an ‘unconscious’ or ‘unofficial’ way, making the transition from knowledge into action difficult (Lambrechts et al. 2013).

What emerged was the idea to create a curriculum with the blend of explicit content knowledge and purposeful pedagogical structure that was partially student led but faculty supported. A successful implementation of a curriculum within one school of education could generate momentum for other schools of education to follow suit.

In order to follow a comprehensive approach to integrate much of what is known about how educational institutions change to become more relevant, we examined the conception of a student led curriculum through a framework that analyzes the process of educational change to advance global education through five perspectives: cultural, psychological, professional, institutional, and political (Reimers 2020).

### A Cultural Perspective of a Student Led Curriculum of Climate Change

A cultural perspective emphasizes that educational practice is the result of shared norms, artifacts and practices which define how education is broadly understood in a society (Reimers 2020). Researchers have found that most people who recycle do not do so out of concern for the environment; they do it because it is socially encouraged – a sociocultural norm (Schultz 2002).

Climate change involves global problems that require social, technological, and political relations to be successful (Räthzel and Uzzell 2009; Wi 2019). CCE also hinges upon a shared vision defined by innovative, multilateral relationships among faculty, students, and surrounding communities (Brundiers et al. 2014). Establishing a shared sense of environmental responsibility is a critical cultural norm, serving as the foundation for numerous sustainability initiatives.

Another way to introduce an inclusive culture of sustainability is to land on a shared meaning of terminology. CCE is inundated with terminologies that are similar in nuance but different in meaning (Varga et al. 2007) which can be intimidating and confusing. Moreover, global standards and guidelines on a common CCE have yet to be implemented.

### A Psychological Perspective of a Student Led Curriculum of Climate Change

Next, a psychological perspective highlights the implications of knowledge about how people learn for the process of changing teaching and learning for students, teachers and others supporting instruction (Reimers 2020). This means that education programs need to be purposefully designed, with specific behavioral changes targeted from the outset. It is not enough for students to acquire theoretical concepts but also to be prepared to act, based on sensible decisions for real-world and complex situations (Vega-Marcote and Varela-Losada 2016). This implies that the educational actions should seek the development of specific skills that foster sustainable actions. Wiek et al. (2011) illustrates five basic competencies of education for sustainable development (ESD) that should be combined to reach this aim:

- **Systems-thinking competence:** the ability to collectively analyze complex systems across different domains (society, environment, economy, etc.) and across different scales (local to global), thereby considering cascading effects, inertia, feedback loops and other systemic features related to sustainability issues and problem-solving frameworks.
- **Anticipatory competence:** the ability to collectively analyze, evaluate, and craft rich visions of the future related to sustainability issues and problem-solving frameworks.
- **Normative competence**: the ability to collectively map, specify, apply, reconcile, and negotiate sustainability values, principles, goals, and targets.
- **Strategic competence:** the ability to collectively design and implement interventions, transitions, and transformative governance strategies toward sustainability.
- **Interpersonal competence:** the ability to motivate, enable, and facilitate collaborative participatory sustainability research and problem solving.

Moreover, best practices in education and effective pedagogies must be infused throughout the design of a competency-driven curriculum. We provide a prototype of a competency driven curriculum embedded with the best practices of twenty-first century learning in section 6.3 (stage 5) and in the Appendix B.

Finally, climate apathy is real and students themselves often do not feel the need to learn or take a course on climate change. Psychologists Kasser and Ryan (1996) define four types of motivations propelling individuals towards a goal. Extrinsic motivation as well as intrinsic ones can incite motivation among students. To incite intrinsic motivation and a connection to nature, Hungerford and Volk (1990) introduce the importance of fostering a personal connection to nature and taking ownership of a problem. Extrinsic motivation can be cultivated when elements of CCE are seen to translate into very practical elements in the workforce. In 2017, the sustainability sector saw a substantial increase in employment as well as average wages $5000 above the national median (Environmental Defense Fund and Meister Consultants Group 2017).

### A Professional Perspective of a Student Led Curriculum of Climate Change

The professional perspective goes beyond teacher pedagogy and focuses on the structure of roles and institutions in integrating expert knowledge into practice (Reimers 2020). Teachers are an essential pillar in CCE, for they are directly responsible for the teaching and learning process. There is plenty of literature on the impact of teacher-student relationships in the classroom (Forbes and Zint 2010; Roorda et al. 2011; García Bacete et al. 2014), as well as the importance of teachers as role models for the development of environmental literacy (Rickinson 2001; Stern et al. 2010).

Beyond providing relational teaching, instructors play a pivotal role as knowledge building catalysts. To aid in these efforts, instructors can carefully consider the selection of resources made available at higher education institutions that promote professional development initiatives. Instructors often have access to an abundance of institutional resources related to climate change, as universities are often the hub of innovative research and cutting-edge technology (Dyer and Andrews 2011).

Furthermore, there has been rising interest in research and practice about ‘students as partners’ through co-created learning and teaching (Cook-Sather et al. 2014; Dunne 2016; Mercer-Mapstone et al. 2017). This bilateral relationship often results in greater learning outcomes and skill enhancements for students.

### An Institutional Perspective of a Student Led Curriculum of Climate Change

An institutional perspective focuses on the educational structures, norms, regulations, and organizational design (Reimers 2020), exploring the work of teaching and learning through a systemic lens. Numerous challenges and internal/external pressures exist for schools of education to bring forth change on a systemic level.

While CCE is important to all education levels, from primary schools to universities ( Harker-Schuch 2019), higher education marks itself distinct in its role in deepening knowledge (Radaković et al. 2017; Vettori and Rammel 2014), inspiring scientifically rigorous expertise (Anderson 2012), and teaching the skills of integration, synthesis, and systems-thinking to cope with complex problems in confronting sustainability challenges (Stephens et al. 2008).

As institutions, schools of education should understand their role in translating cutting-edge research and best practices in education into information that policymakers can apply (Dyer and Andrews 2011). Furthermore, institutions should provide support to grassroots initiatives in SD, for without its support, innovations are short-lived and unable to last beyond personnel and changes within (Hopkins and McKeown 2014).

### A Political Perspective of a Student Led Curriculum of Climate Change

A political perspective recognizes that education affects the various interests of many different groups that are often in conflict with one another (Reimers 2020). The role of universities is especially important in areas of the world where strongly opinionated leaders and media outlets have created confusion among the public about environmental issues like climate change. The topic of climate change resonates with deeply held values, such that adults respond by protecting their group identity and ways of life (Monroe et al. 2017).

In the United States, there is a considerable difference between the probability of taking at least one climate-change related course at public research universities with Democrat-controlled state legislatures versus Republican or split-controlled state legislatures (Hess and Collins 2018). The effects of politics trickle down into teacher and student readiness and belief in teaching and learning about climate change. Stevenson et al. (2016) note that a teacher’s belief that global warming is happening appears to predict a student’s belief.

Despite political challenges, higher education institutions can and must continue to provide an important source of countervailing institutional power to misinformation and lack of policy support when it comes to climate change.

## A Case Study: Implementations of a Student Led Curriculum at the Harvard Graduate School of Education (HGSE)

There were three main reasons to use Harvard Graduate School of Education (HGSE) as a pilot school to test the concept of a student led curriculum.

- First, at the time of writing we had been graduate students at HGSE, allowing us easier access to the resources, staff, and administration needed to design a student driven curriculum within a higher-ed institution.
- Second, the intent of our curriculum and our efforts to scale aligned with the sustainability mission of the university. Harvard University operates with a mission to institutionalize best practices in sustainable operations and translate research and teaching into practice by using the campus to pilot innovative solutions that can be widely replicated (Harvard Office for Sustainability 2016). HGSE, which is celebrating its centennial year in 2020, resides within the greater institutional framework of Harvard University.
- Third, HGSE provides a space where educators from multiple sectors can convene to generate knowledge that improves learning outcomes (Harvard Graduate School of Education 2020a, b), allowing the ideal mix of students who would be taking a curriculum offered at HGSE.

### Methodology

**Fig. 6.1:**
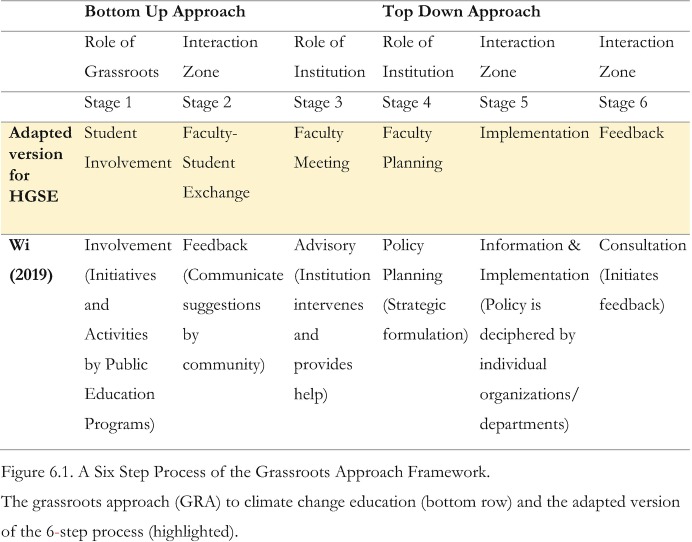
A process for co-creation of a climate change curriculum between university students and faculty

Existing literature points out the fruitful and productive partnerships of grassroots initiatives with large scale institutions and/or government organizations (Reeves et al. 2014; Fadeeva et al. 2014). Wi (2019) lays out a 6-stage process of interaction between grassroots organizations and government agencies within a cycle of a climate change education policy adoption. We have adapted this framework to lay out the steps of the co-creation and collaboration of student and faculty within the cycle of a climate change curriculum implementation. Figure 6.1 juxtaposes the adapted 6 stages with the initial one.

The six stages include:

- Stage 1: Student Involvement: The Role of Grassroots Innovation
- Stage 2: Faculty-Student Exchange: Interaction Zone
- Stage 3: Faculty Meeting: The Role of the Institution
- Stage 4: Faculty Planning: The Role of the Institution
- Stage 5: Implementation: Interaction Zone
- Stage 6: Feedback: Interaction Zone

A. Stage 1. Student Involvement (Role of Grassroots)
  The ultimate goal of the course is to produce graduates from schools of education worldwide who can serve as leaders in the twenty-first century global movement on the pressing issue of climate change mitigation, adaptation, impact reduction and early warning.
  The theory of action on which this proposal is based is:
  **If** a curriculum on climate change is implemented and offered for a minimum of 3 years at HGSE **and** a substantial number of students show interest in enrolling, **then** the course will produce numerous graduates active in various education sectors that have the knowledge, skills, and attitude to help mitigate climate change.
  **If** some HGSE graduates become transformative leaders in education **and** the innovative teaching solutions can be widely replicated (Harvard Office for Sustainability 2016), **then** the course can impart knowledge, skills, and values to the broader community and serve as the blueprint for other education schools.
  More specifically, our curriculum set out to attempt the following objectives:
  First, to help students develop knowledge on climate change and the role of different education sectors and educators in overcoming the issue.
  Second, to ingrain the value of being active agents of change, planning and acting to mitigate climate change.
  Third, to build skills and competencies in collaboratively solving problems related to climate change through education.

B. Stage 2. Faculty-Student Exchange (Interaction Zone)
  Previous efforts to reorient teacher education to address sustainability and innovations related to ESD were carried out on a personal scale and were short-lived, unable to last beyond personnel and changes in innovation (Hopkins and McKeown 2014). It became evident that our curriculum would also need sustainable support from the institution.
  We approached a professor at HGSE, a cognitive scientist whose work centers around ecology, climate change, and the use of virtual and augmented reality in the classroom. In particular, her research identifies ways in which understandings about the nature of causality impact our ability to deal with complexity in our world. Her extensive expertise and experience on the topic made her our top candidate as we approached her with the idea of implementing a climate change curriculum at HGSE.
  This exchange indicated that for a curriculum to be introduced at HGSE, we would have to identify and demonstrate the need for a course on climate change. If and only then, faculty could move forward with the motion, and the course would be examined for approval. To aid that process, we administered a needs assessment to demonstrate the existing gaps between current course offerings and student demand for the intended curriculum. Data we collected could paint a better picture of the status quo.
  i. Survey Administration and Interpretation
    In order to assess preliminary needs and interest in a climate change curriculum for the twenty-first century at HGSE, we administered a Knowledge, Attitude, and Practice (KAP) survey to 66 respondents comprised mostly of HGSE students. A randomized sample was collected through an online survey soliciting students within HGSE and the greater Harvard community. Limitations of the survey include probable bias towards climate activism due to its opt-in design, as well as its small sample size, representing less than 10% of the overall HGSE student population. The 66 participants were affiliated with various programs at HGSE and planned to go into different sectors in education, reflecting the diverse student composition in education sectors that our curriculum targets. Each question was designed specifically to correspond with a Knowledge, Attitude, or Practice (KAP) assessment of student’s understanding and interest of climate change. (Table 6.1).

**Table 6.1:** KAP survey to understand HGSE student’s interests of climate change

	Question	Respondents
Affiliation	State your school affiliation	Among 66 graduate students within the wider Harvard network, 94% of the sample (62) were comprised of students at the Harvard Graduate School of Education
	Which education sector do you plan on going into after graduation?	(33.3%) Non Profit Organization (17.5%) K-12 School System (12.7%) Media/Technology (12.7%) Consulting (7.9%) Government (6%) Higher Education (9.9%) Other
Knowledge	How would you rate your knowledge about climate change? Self-assessed knowledge on ESD	(66.2%) I have gathered some information about the subject. (16.9%) I have been studying the effects of climate change (15.4%) Very limited; I have heard about it, but I am not aware of the fact (1.5%) I am an expert on climate change
	What do you think is the cause of climate change?	22 out of 66 respondents (33.3%) replied correctly
	What are some of the direct effects of global warming?	29 out of 66 respondents (43.9%) answered correctly *13 respondents (19.7%) answered correct for both questions assessing current knowledge of climate change
Attitude	Do you think climate change is happening?	(90.5%) Very certain (7.9%) Somewhat certain (1.6%) Not very certain
	How concerned are you about climate change?	(64.6%) Very concerned (30.8%) Somewhat concerned
	How big of a role do you think *education* plays in mitigating climate change?	(64.6%) Very big (30.8%) Somewhat big (3.1%) Not very big (1.5%) Not at all big
	How big of a role do you think *educators* plays in mitigating climate change?	(49.2%) Very big (35.4%) Somewhat big (13.8%) Not very big (1.5%) Not at all big
Practice	What are some efforts you’ve undertaken to mitigate climate change?	(80.7%) Reducing one’s own carbon footprint (including taking public transportation, recycling, reducing plastic use, buying sustainable products, limiting the consumption of meat, etc.) (11.5%) Raising awareness (sharing information with families and talking to coworkers regarding the issue etc.) (7.7%) Engaging in political action (voting for leaders who demonstrate concern for climate change, putting pressure on leadership to encourage compost, and joining climate strikes) (5.8%) No effort (3.8%) Skepticism of individual efforts in mitigating climate change
	What are some efforts you’ve undertaken to mitigate climate change *through education*?	(25.5%) Informal conversations (23.4%) Teaching students (6.4%) Using social media (6.4%) Organizing events (6.4%) Advocacy (6.4%) No attempts
Interest	Would you be interested in taking a course at HGSE on climate change and education?	(52.3%) Yes (47.7%) No
    - Knowledge
      A survey of the participants’ self-assessed knowledge on climate change revealed 66.2% of the respondents reporting they had gathered some information about the subject, and 15.4% claiming to have very limited knowledge on climate change. To test the actual knowledge of climate change, two multiple-choice questions were administered, questioning the causes and effects of climate change. Only 13 respondents, comprising 19.7% of the total sample answered correctly on both questions, demonstrating a significant gap in students’ *actual* knowledge of climate change.
    b. Attitudes
      90.5% of the respondents were very certain that climate change was happening, showing a strong consensus on the existence of the phenomenon. 95.4% of respondents expressed concerns regarding climate change, with 64.6% of the respondents responding that they were very concerned about climate change. There was a general consensus with regards to whether *education* had a significant role in mitigating climate change, with 95.4% of respondents selecting 4 or 5 on a scale of 5, with higher numbers indicating greater significance. Strong agreement (84.6% of respondents who answered a 4 or 5 on a scale of 5) was also observed in responses to the question of whether *educators* were significant in mitigating climate change.
    c. Practice
      By analyzing qualitative responses to questions asking one’s efforts taken to mitigate climate change, we could extract five general themes that represented the responses: reducing one’s own carbon footprint, raising awareness, engaging in political action, no effort, and skepticism of individual effort in mitigating climate change. The majority of the respondents (90.4%) have made efforts in mitigating climate change, with the most common responses related to reducing their own carbon footprint (80.7%).
      Next, an analysis of one’s attempt to mitigate climate change through education yielded six different themes: informal conversations, teaching students, using social media, organizing events, and advocacy. The most common attempts were engaging in informal conversations (25.5%) and teaching students on the topic of climate change (23.4%).
    d. Interest
      Thirty-four students, or 52.3% of the total number of respondents, showed an interest in taking a course on climate change and education.
      Whereas needs assessments like the one we conducted are not meant to be generalizable nor predictive of actual behavior, this figure provides some indication that there is considerable interest in CCE among students at the Harvard Graduate School of Education. We shared the results of the survey with the academic leadership of the school, and the feedback was immediately positive. The urgency of the situation and the demand from the student population clearly demonstrated the need for this class.

C. Stage 3. Faculty Meeting (Role of Institution)
  Creating new classes can be a time-consuming effort that requires several layers of review within the faculty and by a governing body that controls curricular and academic changes. Such a review process typically requires creation of a syllabus and reading list (Appendix A) for the course as well as examining the credentials and expertise of those who would teach the course to show they have adequate expertise.
  We waited for a follow-up from faculty, and much to our delight, were informed that such a course was approved.

D. Stage 4. Faculty Planning (Role of Institution)
  Each institution charts out a curriculum implementation process, systematically organizing what will be taught, who will be taught, and how it will be taught. At HGSE, there is a three-step process that aids faculty in planning a course curriculum.
  Phase 1: One on One Conversation
  Phase 2: Course Preparation
  Phase 3: Teaching Team Meeting.
  i. Phase 1: One on One Conversation
    In this phase, faculty reviews the course planning guide and forms a teaching team. Together, they discuss and agree on due dates.
  ii. Phase 2: Course Preparation
    In the course preparation phase, design elements of the course are carefully considered. The scope (breadth of knowledge, skills, attitudes, and behaviors) and the sequence (order) of the course would be discussed. Appropriate materials, pedagogy, and effective class activities are chosen within the design of the curriculum (Brundiers et al. 2014). Formative and summative evaluation methods are examined which will measure the effectiveness of the curriculum.
    Faculty members are supported by the Harvard Graduate School Course Planning Team. The Teaching and Learning Lab provides faculty support through individual consultations and group professional opportunities to craft intelligent and thoughtful course designs as well as producing instructional resources (HGSE Teaching and Learning Lab 2020).
  iii. Phase 3: Tea+ching Team Meeting
    Faculty assistants, faculty, and teaching fellows are determined and meet to discuss the facilitation of the class.

E. Stage 5. Implementation (Interaction Zone)
  The authors participated in the faculty driven process of the implementation by offering assistance and support in the ideation and design of an ESD curriculum aimed at the school of education. Two prototype lessons of the curriculum can be accessed in the Appendix B.
  i. Standards
    Our curriculum seeks to meet standards provided by the 2030 Sustainable Development Goal, target 13 Climate Action. In particular, goal 13.3 focuses on improving education, awareness-raising, and building institutional capacity related to climate change mitigation, adaptation, impact reduction and early warning. Furthermore, we have aligned our curriculum to accommodate the four pillars of HGSE foundational elements of Learning, Development, and Teaching; Evidence; Equity and Opportunity; Organizations and Systems (Harvard Graduate School of Education 2020a, b). In order to emphasize the importance of making knowledgeable behavioral decisions, we included the five basic competencies as defined by Wiek et al. (2011): Systems thinking competency, Anticipatory competency, Normative competency, Strategic competency, and Interpersonal competency.
  ii. Format
    *Climate Change for Educators* will be a 12-week semester long course, comprised of two 90-min classes focusing on a weekly theme. We based the overarching framework of the curriculum design using the UN SDG aligned curriculum *Empowering Students to Improve the World in Sixty Lessons* (Reimers 2017) in addition to *Becoming Global Thinkers: Thinking about Distant Causes and Effects, Causal Learning in the Classroom (CLIC)* (Grotzer et al. 2015).
    Furthermore, our lesson plan prototype has been designed so that each of the weekly topics and units can function independently of one another. Thus, they can be separated and blended into existing structures of curricula units to impart knowledge, skills, and values that teach educators about the critical issue of climate change.
  iii. Pedagogical Design
    - Knowledge Transfer
      In 1956, Bloom categorized cognitive learning objectives in a progressive hierarchy from least to most complex levels which include: knowledge, comprehension, application, analysis, synthesis, and evaluation. Based on his taxonomy, this curriculum devotes the first class of each week building upon basic, fundamental skills such as ‘knowledge,’ and ‘comprehension’ via lectures and engaging discussions. A knowledge-based start will help to foster an atmosphere of intellectual discourse in the classroom (Fook 2012), and specifically of accumulating, deepening, and transferring knowledge of the environment to a large mass of educators (Radaković et al. 2017).
      After a short lecture, faculty will engage students in a lively discussion, which will foster student engagement.
    b. Engagement
      Lectures are followed by an invigorating student led discussion, for students to ‘synthesize’ (Bloom 1956) what they have learned. Discussions help bring out the importance of engagement and fostering a culture of student-centered learning (Anderson 2012). Discussion prompts will center around the essential questions posed by faculty, and this active facilitation is enhanced as faculty demonstrate the changing role of a teacher; from “content expert” to “curriculum facilitator,” in this new era of learning (Godsey 2015). Active listening is encouraged, and main points are organized on a board framing the argument and building insights.
    c. Cross-Disciplinary
      Climate change education inevitably requires having to incorporate a blend of multi-disciplinary academic subjects (Lindblom-Ylänne et al. 2006; Lueddeke 2003; Nevgi et al. 2004; Singer 1996). Topics within the scope of this curriculum include socio-political issues surrounding the scientific facts of climate change, innovative technology serving as possible solutions, as well as the role of education in mitigating climate change. Week 3 will touch upon the cognitive function of how people learn, with regards to understanding that climate change requires an ‘action-at-a-distance’ approach. Weeks 4–11 will cover a myriad of ways in which education can help mitigate the impact of climate change. Some topics include curriculum design, professional development of teachers, school operations, informal education, measurements, education policy, and climate justice. In the final week of class, students will give their presentations and share the takeaways from the course.
    d. Project Based
      The second class each week will consist of ‘applying,’ ‘synthesizing,’ and ‘evaluating’ (Bloom 1956) what they know via engaging projects. Numerous studies have highlighted the benefits of active, project-based learning (Leigh 2009). The semester-long project involves a group of 3–5 students (Henke 1985) with varying levels of experience in multiple education sectors, collaborating to craft a holistic educational strategy to mitigate climate change. Students are asked to formulate project groups around a jurisdiction with meaningful personal ties, helping to contextualize learning as local, tangible, and personally relevant (Cone et al. 2012; Anderson 2012). Each jurisdiction will highlight the different agenda and perspectives present today in the twenty-first century global climate change movement. Students will give presentations in the final week and submit a 20-page-paper as a final project. Throughout the project, students are active participants in their own learning which will include the design of their experiences and the realization of their learning outcomes. Ultimately, students can take full ownership of their own learning. This class will involve weekly group assignments (Monroe et al. 2017) that students will start in the class but finish as collaborative homework.
    e. Situated
      The second half of the week additionally focuses on student-centered activities, guest lecturers, simulations, and excursions to provide a thought-provoking experience for students to experience real life perspectives in the topic of climate change. In week 7, students will take an excursion to a nearby environmental institution. With multiple studies revealing the importance of making the distant threat of climate change personally relevant and meaningful (Shome et al. 2009; Fook 2012; Moser and Dilling 2007; Wibeck 2014), excursions can help make the threat of climate change real, tangible and immediate (Cone et al. 2012). We also bring in local guest speakers committed to the field to bring expert knowledge into the classroom (Leigh 2009; Theobald et al. 2015) serving as a relevant local source of inspiration to the students all the while minimizing carbon footprint expenditure.
    f. Real World Situations
      Our audience are adult learners, who see themselves as capable of self-direction and incentivized by tasks that will prepare for social and occupational role competency (El Sawi 1996). Each week, we provide assignments that carry out practical exercises that encourage learners to put into practice the theories they learned. Our adult learners have the autonomy to carry out their given task, on very practical elements that can be utilized in the workforce.
    g. Faculty Involvement
      Students will be encouraged to meet with instructors at least once every 3 weeks so that expectations and standards from both sides of the teaching team and the students are well integrated, coherent, and harmonized. Intimate feedback from the teaching team enhances student learning, and the instructor takes away with a solid knowledge and understanding of student’s progress.
    h. Accountability
      Students will be assessed on a weekly basis, with weekly projects consisting 60% of the total grade, and the semester long final projects being worth 40%. As students are working in groups, accountability measures such as self-assessments and peer-evaluation sheets will be collected by every member of the group. Student projects would be assessed via a rubric.

F. Stage 6 Feedback
  In this final stage, student-faculty interaction is oriented towards the students who are taking the course and the faculty teaching the course. Feedback from students in the form of formative and summative evaluations is collected to improve and sustain the curriculum.

## Implications & Conclusion

Our chapter described and analyzed efforts of implementing a climate change curriculum within the Harvard Graduate School of Education (HGSE) that helps to build competencies for potential leaders in different education sectors so that they can combat climate change collaboratively. However, our goal is not the development and implementation of a single curriculum. We aspire to achieve a collective legacy of preparing educators within the whole-of-system and achieve widespread change, which will require concentrated and concerted efforts to disseminate the importance of climate change curricula at multiple schools of education.

There are obvious limitations in focusing solely on a curriculum without simultaneous efforts to promote sustainability as endorsed within a whole-school model of ESD within the entire Harvard Graduate School of Education. Moreover, the disruption of Covid-19 may have significant, damaging effects to the immediate implementation of the curriculum, as well as its aftermaths in the upcoming years should there be a shift to a digital model of education. Furthermore, the institution shift into a mode to mitigate the impact of the pandemic on the school may induce several entanglements previously unanticipated in the conception of the curriculum.

To answer our original question: If students played an equal role in the development of curricula, selection of course content, and initiation of applied projects, how different might the impact of the academy become?

Our curriculum explored the successful beginnings of a student/faculty co-creation and collaboration. This is not to say there were not tensions between expert and novice and between discovery and direction. For a successful blend of bottom-up and top-down interaction, multiple components must come together under mutual respect, common understanding, and shared responsibility. Learner agency, increased satisfaction with the academy, and heightened engagement came with the author’s participation of a curriculum design. The institution was provided with insights into students’ perspective and needs as well as enhanced capacity to promote a holistic engagement with learners.

Ultimately, a whole-of-systems approach to climate change means that the entirety of the system promotes action for reducing climate change, sharing the responsibility within all. While each individual component of these five perspectives is integral to creating a culture of change, it is the sum of the respective perspectives and the interaction between all of the elements that will be critical in the 21st global movement of shared environmental responsibility. And a curriculum like ours, educating future leaders within schools of education, is a step closer in the right direction of attaining a sustainable future for all.

## Appendices

### Appendix A. Syllabus

**Table:** Class title: Climate change for educators

	Lecture classes	Project based classes	
Week 1	Class 1 Introduction to climate change education	Class 2 Activity #1: Formation of project groups.	Building up a background knowledge of climate change
Week 2	Class 3 Socio-political issues surrounding the scientific facts of climate change	Class 4 Guest lecturer on solar engineering Activity #2: Innovative technology	
Week 3	Class 5 What makes climate change hard to accept?	Class 6 Guest lecturer EcoMuve Activity #3: Ethics and individual impact	
Week 4	Class 7 Whole-of system model to climate change education	Class 8 Guest lecturer: GSD professor Activity # 4: Identifying sustainable school operations	Climate change within local school systems
Week 5	Class 9 Supporting teachers to teach	Class 10 Activity # 5: Designing ProD activity and teacher support systems	
Week 6	Class 11 What makes for a good climate change curriculum?	Class 12 Activity #6: Creating a curriculum based on competencies and best learning practices	
Week 7	Class 13 Informal education – Educating the mass	Class 14 Excursion. Climate change in the real world. Visit the Harvard Museum of Natural History: Climate change exhibition. Activity # 7: Design an awareness campaign to draw in the audience.	
Week 8	Class 15 Economic metrics, measurements, and evaluations	Class 16 Activity # 8: Find measurements of HGSE operations and assess its energy consumption.	
Week 9	Class 17 Climate change education in the United States	Class 18 Activity # 9: Write a policy memo	Climate change in the world
Week 10	Class 19 The state of global climate change education	Class 20 Climate change simulation – world climate	
Week 11	Class 21 Climate justice and ethics: Migration, resilience, and adaptation	Class 22 Review class Activity # 10: Prepare for a 10-min final presentation	
Week 12	Class 23 Presentations of projects &wrap up.	Class 24 Presentations of projects &wrap up. Activity # 11: Final paper	Review

### Appendix B. Prototype Lessons of a CCE Curriculum at HGSE

Week 1 Class 1

Introduction to Climate Change Education

***Knowledge & Application***

**Time:** 90 min

**Standards:** Sustainable Development Goals 13.1, 13.3; HGSE foundational element: Learning, Development, and Teaching; Evidence; Equity and Opportunity; Organizations and Systems

**Competencies:** Systems-thinking, Anticipatory, Normative Strategic, Interpersonal

**Summary/Rationale:**

The 2030 Agenda for Sustainable Development, adopted by all United Nations Member States in 2015, provides a shared blueprint for peace and prosperity for people and the planet.

Sustainable Development Goal 13 asks member states to take urgent action to combat climate change and its impacts. Specifically, target 13.3 implores nations worldwide to improve education, awareness-raising, and human and institutional capacity on climate change mitigation, adaptation, impact reduction and early warning. Achieving this legacy goal requires involving various educators, agents in delivering education and raising awareness. This course will help explore how a climate change curriculum at the Harvard Graduate School of Education can prepare educators to mitigate climate change.

**Instructional Goal:** Students are provided with a general overview of climate change education and the important role of educators within education for sustainable development.

**Student Learning Objectives**
- Cognitive:
  - The learner understands that the anthropogenic emission of greenhouse gasses account for almost all the increase in greenhouse gasses in the atmosphere over the last 150 years.
  - The learner understands the role educators can assume in the shared responsibility of environmental sustainability.
  - The learner knows the five competencies required in climate change education.
- Socio-Emotional
  - The learner can recognize that the protection of the global climate is an essential task for everyone and the need to re-evaluate our worldview and everyday behaviors.
  - The learner understands the importance of a whole-of-systems model, in its interaction and support.
- Behavioral Learning
  - The learner is able to communicate and endorse positive approaches to CCE.

Essential questions:

- *How does the role of education and educators fit in the global movement of climate change?*
- *What is climate change and its causes? How does human play a part in climate change?*
- *What is the purpose of climate change education (CCE)?*
- *How can we approach CCE so that knowledge can be transpired into action?*

Sequence of activities

1. (30 min) Introduce the course

Faculty welcomes students and introduces themselves. He/She will then go over the course format, pedagogical choices, and the scope of the curriculum. Faculty will model best practices of teaching and learning both in pedagogy and content.

Something you might say,” I will go over course logistics and sketch out the main issues in a very preliminary way. Students(you) should feel free to raise questions of each other or of me.”

2. (35 min) Lecture – Knowledge transfer

Faculty may want to introduce a thought-provoking video on CC or state of CCE to spark this lecture. Briefly explain the key points of the readings students have been asked to complete for homework.

(Video: National Geographic https://www.youtube.com/watch?v=QwLyscT3NgI)

- Introduction to Climate Change

*Brief overview of history of Earth’s atmosphere: what is a greenhouse gas?*

Students should have completed the following readings for homework:

Reading: Allégre & Schneider, Scientific American, 2005. The evolution of Earth

Reading (optional): Meteorology Today, Chapter 18, Earth’s Changing Climate

(b) Climate Change and Education − Importance of Educating Educators Reading: https://oxfordre.com/environmentalscience/view/10.1093/acrefore/9780199389414.001.0001/acrefore-9780199389414-e-56
(c) What are Competencies in CCE and why is it important? What are some tensions seen in CCE today?
  Reading: Vega-Marcote, Pedro & Varela-Losada, Mercedes. (2016). Basic Teacher Training Oriented Toward Sustainability: Why and How to Carry It Out Today?
(d) A Whole-of Systems model in CCE/ESD.
  Reading: Ferreira, Jo-Anne & Ryan, Lisa & Tilbury, Daniella. (2007). Mainstreaming education for sustainable development in initial teacher education in Australia: A review of existing professional development models. Journal of Education for Teaching.

3. (25 min) Discussion – Applying what you know.

This last 25 min is reserved for a whole-class reflection. Active, engaged student participation is important to maximize learning outcomes.

Essential question: How does the role of education and educators fit in the global movement of climate change?

There are several ways you can set up this discussion.

1. A whole-class discussion in which discussions are freely encouraged
2. A case study discussion – Encourage students to think about the prompt by providing a specific case of CCE. As students discuss the prompt, organize their ideas on the board through a framework to bring out further insights and innovative approaches.
3. Negotiation/Simulation – Provide students with a designated ‘role.’ i.e. teacher, student, administration, government etc. Allow students to explore this discussion through the lens of the role they are given.

Discussion should be primarily student led, with faculty facilitating the discussion by bringing out diverse voices (with the occasional cold calling) and probing students to capture relevant knowledge and nuances of the case.

Week 1 Class 2

Activity #1 Formation of Groups

***Application & Evaluation***

**Time:** 90 min

*Project*

**Standards:** Sustainable Development Goals 13.2 HGSE foundational element: Learning, Development, and Teaching; Organizations and Systems

**Competencies:** Systems-thinking, Interpersonal, Normative, Anticipatory

**Summary/Rationale:**

Every 2nd half of the week, classes are centered around a semester long project, involving a group of 3−5 students (Henke 1985) with varying levels of experience in multiple education sectors, collaborating to craft a holistic educational solution to mitigate climate change.

Students will formulate their own groups based on personal interests and goals, but the groups must be centered around a common geographical tie. The jurisdiction of their choice will highlight the different agendas and perspectives present today in the twenty-first century global movement of sustainable development. As students pick a jurisdiction with meaningful personal ties, this helps to contextualize climate change learning as local, tangible, and personally relevant (Cone et al. 2012; Anderson 2012).

Each week, groups are given structured activities that allow members to explore a different perspective of CCE, using systems thinking to approach the multi-layered movement. Through class presentations and group assignments there will be ample opportunities for peer-learning and cultivating global citizenship. Students will give presentations in the final week and submit a 20-page-paper as their final project.

**Notes:** Make sure students understand “group dynamics” and go over the “key mistakes of forming groups” slide. It is important for the class to understand how to select group members and to function well together throughout the semester.

——————————————————————

**Student Learning Objectives**
- Cognitive
  - The learner can discern that different jurisdictions face different dilemmas concerning climate change.
- Socio-emotional
  - The learner is able to encourage others to protect the climate.
  - The learner learns to work in a group setting.
- Behavioral
  - The learner engages in cooperative team dynamics.

——————————————————————

**Sequence of activities**

1. (5 min) Introduce the activity of the day

Explain to the class the expectations for classes in the 2nd half of the week. We will establish norms for this ‘project-based activities’ class.

Introduce the semester long project by starting with the goal and objective. Their primary goal for this class is to form project groups they will be working with for the remainder of the semester.

2. (30 min) Step-by-step approach to applying concepts into projects.

**Step 1.** How does ‘geography’ play a factor in climate change education? How does the country/region/political affiliation/organization you belong to/identify with shape your perspective on climate change/environment?

Reading Assignment:

https://www.climateinteractive.org/programs/world-climate/facilitator-resources/

Skim the 6 briefing statements of: The United States, European Union, Other Developed, China, India, Other Developing.

PowerPoint slide: How might geography/culture influence my view?

- Developed Nation: (Pro Climate Change) European Union, Canada, Japan, South Korea (Majority of OECD member states)
- Developed Nation: United States (Political affiliations in the United States)
- Rapidly Emerging Nations: China, India
- Developing Nation: Ghana, Myanmar
- Pacific Island Nation: Maldives

Allow students to take 5 min to reflect on the jurisdiction they would like to focus on throughout this semester. Make sure students are aware that the groups they choose in this class may be difficult to change throughout the semester.

**Step 2.** Working in Groups:

Reading Assignment-

*Edmondson, A. C. (2012) Teaming to learn, innovate, and compete. In Teaming: How organizations learn, innovate, and compete in the knowledge economy. San Francisco: Jossey-Bass. Chapter 2, pp. 45−80*

PowerPoint Slide-

*What are some common misconceptions to forming groups?*

1. More is better, so make the team big → Optimal group size: 4.6
2. Similar people will get along better → Heterogeneity: variety of skills and backgrounds works better
3. Everyone understands how to work in a group → Not so! You must develop shared understanding of how to work in a group; Don’t forget to keep looking outward – for new sources, new inspirations; Don’t ignore an “off-target” comment … it may reflect an essential perspective from a different group; Teamwork can be stressful: “Serious work means serious tensions”; Fear of not getting along, Fear of being “wrong,” Silence is easier than speaking

*What are positive team behaviors?*

1. Everyone has an obligation to participate
2. From brainstorming forward, build on others’ good ideas
3. Respond honestly – doesn’t help the group to withhold what you really think
4. Critique ideas; not individuals – make the team a psychologically “safe space”
5. Be flexible
6. Seek consensus … … and have a process for making decisions
7. Share leadership … … but define clear roles for specific tasks and stages
8. Minimize egos … … and maximize fun and humor’

Conduct an online survey- Ask students to complete a brief survey of one’s strengths, interests, and competencies.

Results will be displayed on the screen, and the class can see the unique skillsets each members of the class possess.

**Step 3.** Formulate your team.

*3−5 members to a team based on regional interest, and skills assessment, students are asked to move around to find members.

3. (30 min) Meeting the Team

Faculty and TFs go around to make sure groups are working well.

The main task is allowing students to formulate their groups for the semester. Once small groups begin to congregate, facilitate the session by asking members to introduce themselves, their interest in a geographic sector and for this course overall.

*Note that students will be adding/dropping the course until the add/drop deadline.

——————————————————————

**Assignment:** Write up a short reflection about:

1. What are some group norms you tend to establish, follow, and promote?
2. Why you are interested in this particular jurisdiction?
3. What you hope to learn and what questions you hope to answer.

You are welcome to write a few short paragraphs, or draw up an outline, or list a few bullet points. The goal is to set a brief agenda for the semester, generate ideas, and establish expectations.

While the first assignment is an individual reflection, the majority of assignments are group driven, and students are encouraged to meet outside of class time for the purpose of the projects.
